# Methylation regulation for FUNDC1 stability in childhood leukemia was up-regulated and facilitates metastasis and reduces ferroptosis of leukemia through mitochondrial damage by FBXL2

**DOI:** 10.1515/med-2023-0810

**Published:** 2024-06-27

**Authors:** Sihai Tan, Yirong Ge, Jing Bi

**Affiliations:** Department of Pediatric, Hubei Enshi Tujia and Miao Autonomous Prefecture Central Hospital, Hubei Province, Enshi 445000, China

**Keywords:** FUNDC1, FBXL2, ubiquitination, ferroptosis, methylation

## Abstract

Leukemia, the most common malignant tumor in childhood, can be categorized into acute leukemia and chronic leukemia. However, the role of FUNDC1 in childhood leukemia (CL) remains unknown. This study aims to investigate the effects of FUNDC1 on patients with CL and its underlying mechanism both *in vivo* and *in vitro*. The mRNA expression levels of FUNDC1 were found to be up-regulated in serum samples from CL patients as well as in leukemia cell lines. Furthermore, it was observed that the mRNA expression of FUNDC1 was lower in stage I–II CL patients compared to stage III–IV patients. The up-regulation of FUNDC1 was found to promote leukemia metastasis. Additionally, it was discovered that FUNDC1 up-regulation reduces ferroptosis by inhibiting mitochondrial damage. In a leukemia model, FUNDC1 up-regulation induces the expression of FBXL2. Moreover, FUNDC1 up-regulation reduces FBXL2 ubiquitination, thus maintaining FBXL2 protein expression in leukemia. By inducing FBXL2, FUNDC1 reduces ferroptosis in leukemia through the inhibition of mitochondrial damage. The stability of FUNDC1 is controlled by METTL3 methylation. Overall, this study sheds light on the role of FUNDC1 in CL and provides insights into its underlying mechanisms.

## 1 Introduction

Leukemia, the most common malignant tumor in childhood, can be classified into acute leukemia (AL) and chronic leukemia [1]. Among children, AL is more prevalent, with 80% of cases being acute lymphoblastic leukemia (ALL) [2]. In China, the incidence of childhood leukemia (CL) is approximately 4/100,000, and it is increasing by 9,000 cases per year. Leukemia has a poor prognosis and is a leading cause of death in children [3]. Therefore, understanding the epidemiological characteristics of CL is crucial for early diagnosis and treatment [3].

Leukemia is the most common malignant tumor disease in childhood. With the continuous progress of medical technology, the 5-year survival rate of leukemia patients has exceeded 80%. Leukemia develops into a chronic disease, and the number of cancer survivors increases accordingly [1]. China usually defines cancer patients who have completed routine treatment and entered a stable follow-up period as cancer survivors [2]. The main social need for cancer survivors in their school-age and adolescence is to return to school. However, these survivors may face the risk of delayed adverse reactions or health restrictions, which will affect the school adaptation of CL survivors [3].

Cell is a crucial process for maintaining normal growth, development, and internal environment stability in individuals. The classical modes of cell death include apoptosis, autophagy, and necrosis [4]. Ferroptosis is a newly discovered form of cell death regulation characterized by cell volume shrinkage, increased mitochondrial membrane density, and reduced cristae [5]. Unlike necrosis and apoptosis, ferroptosis is primarily characterized by the generation of reactive oxygen species (ROS), lipid peroxidation, and iron accumulation [6]. Recent research on the mechanism of ferroptosis has shown its close association with the occurrence and progression of tumors [6,7]. Leukemia and lymphoma, as malignant tumors of the hematopoietic system, have low efficacy with existing treatment plans, necessitating exploration of new treatment models [8]. The advancements in ferroptosis research have provided novel insights for the treatment of hematopoietic system tumors [9].

As one of the core regulatory receptors for damaged mitochondrial clearance, FUN14 domain containing (1) (FUNDC1) plays an important protective role in myocardial injury [10]. FUNDC1 is a protein located in the outer membrane of mitochondria, which participates in mitochondrial fusion and division to maintain heart function. The absence of FUNDC1 can lead to heart failure. Under normal circumstances, FUNDC1 interacts with the mitochondrial fusion protein optic atrophy protein 1 located on the inner membrane of mitochondria to provide energy for myocardial cells [11,12]. Under stress conditions, FUNDC1 can promote the translocation of mitochondrial related protein 1 to the mitochondrial outer membrane, thereby inducing mitochondrial division. FUNDC1 is expressed in various cells, tissues, or organs and can recruit LC3 to regulate cell autophagy through its unique Leucine-rich repeat conserved domain. There is evidence that the abnormal expression of FUNDC1 is involved in the pathological progression of various cancer diseases and can serve as a potential target [11,12]. However, the role of FUNDC1 in CL is unknown. This study investigated the effects of FUNDC1 on patients with CL and its underlying mechanism *in vivo* and *in vitro*.

## 2 Materials and methods

### 2.1 Patients with CL

This study was approved by the Ethics Committee of our hospital. All the serum samples were immediately snap frozen in liquid nitrogen and stored at −80°C for further using. Pathological evaluation was performed according to the WHO classification by two experienced clinical pathologists.

Inclusion criteria were (1) meets the diagnostic criteria related to childhood ALL, (2) age <12 years old, (3) family members have informed consent to the treatment method and related precautions, and have good compliance, (4) complete clinical data, and (5) the resident population of this region.

Exclusion criteria included: (1) intolerance to the medication used in this study, (2) after diagnosis, without treatment or voluntarily giving up treatment or transferring to another hospital during the treatment period, (3) received corresponding treatment before enrollment, (4) concomitant systemic acute and chronic infections, and (5) severe liver and kidney dysfunction.


**Ethical approval:** The current study was approved by the Ethics Committee of Hubei Enshi Tujia and Miao Autonomous Prefecture Central Hospital and complied with the guidelines outlined in the declaration of Helsinki were followed.
**Informed consent:** Informed consent was obtained from all participants.

### 2.2 Cell culture and transfection

HS-5, 6T-CEM, HL-60, K-562, and MOLT-4 cells were performed in compliance with ATCC protocols and incubated in a 5% CO_2_ atmosphere at 37°C. FUNDC1 plasmids (sc-428195, Santa Cruz Biotechnology, Inc.) or si-FUNDC1 plasmids (sc-145273, Santa Cruz Biotechnology, Inc.) were transfected into GC cell lines using Lipofectamine 2000.

### 2.3 Quantitative polymerase chain reaction (qPCR)

Total RNAs were isolated with RNA isolator total RNA extraction reagent (Takara) and cDNA was synthesized using PrimeScipt RT Master Mix (Takara). qPCR were performed with the ABI Prism 7500 sequence detection system according to the Prime-ScriptTM RT detection kit. Relative levels of the sample mRNA expression were calculated and expressed as 2^−△△Ct^.

### 2.4 Immunofluorescent staining

Immunofluorescent staining was executed as literature [1]. Cells was incubated with FUNDC1 (ab224722, 1:100; abcam) and FBXL2 (ab153842, 1:100; abcam) at 4°C overnight after blocking with 5% bovine serum albumin (BSA) for 1 h. Cells were incubated with goat anti-rabbit IgG-cFL 488 or anti-rabbit IgG-cFL 555 antibody (1:100) for 2 h at room temperature, stained with 4′,6-diamidino-2-phenylindole for 15 min, and then washed with phosphate buffered saline for 15 min. The images of cells were obtained using a Zeiss Axioplan 2 fluorescent microscope (Carl Zeiss AG, Oberkochen, Germany).

### 2.5 Proliferation assay and EDU staining

For cell counting kit-8, after 48 h of transfection, a total of approximately 2 × 10^3^ cells/well was seeded into 96-well plate. After culturing at indicated time, the cellular proliferation was detected using CellTiter-GloR Luminescent Cell Viability Assay (Beyotime) according to manufacturer’s instructions.

For ethynyl deoxyuridine (EdU) incorporation assay, EdU (10 mM) was added to each well and cells were fixed with 4% formaldehyde for 30 min. After washing, EdU was detected with Click-iTR EdU Kit and images were visualized using fluorescent microscope (Olympus).

### 2.6 Western blot

Western blot was executed as literature [1]. The membranes were incubated with primary antibodies: FUNDC1 (ab224722, 1:1,000; abcam), FBXL2 (ab153842, 1:1,000; abcam), GSH peroxidase 4 (GPX4) (ab125066, 1:1,000; abcam), and β-actin (sc-47778, 1:5,000, Santa Cruz Biotechnology) after blocking with 5% BSA in tris-buffered saline, followed by incubation with peroxidase-conjugated secondary antibodies (Santa Cruz Biotechnology). The signals were detected with the ECL system and exposed by the ChemiDoc XRS system with Image Labsoftware (Bio-rad).

### 2.7 Statistical analyses

Graphad Prism 6 was used for the statistical analysis. *P* < 0.05 was considered statistically significant. Comparisons of data between groups were followed using Student’s *t*-test or one-way analysis of variance, followed by Tukey’s *post hoc* test.

## 3 Results

### 3.1 Expression levels of FUNDC1 in CL

**Figure 1:**
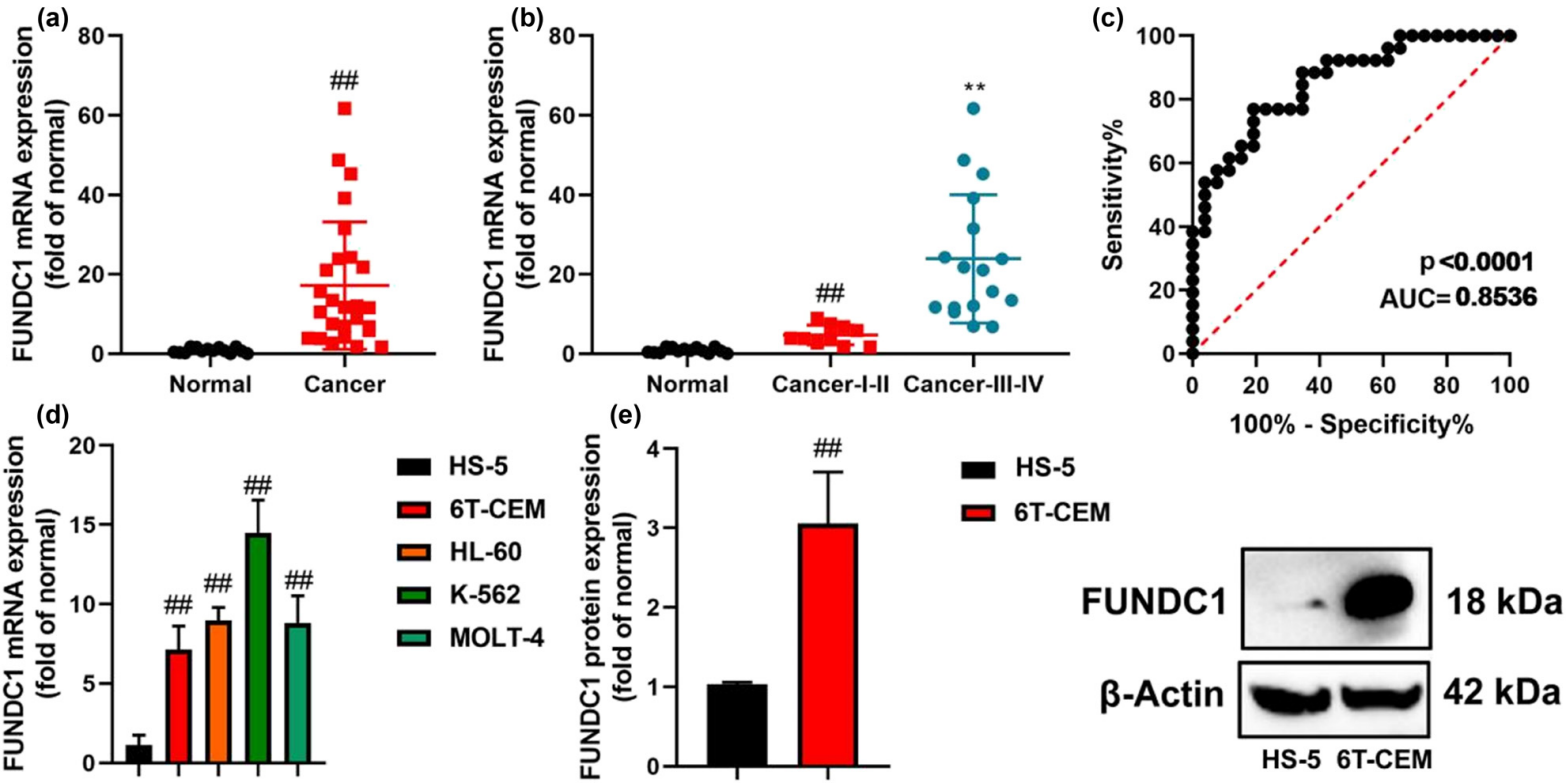
Expression levels of FUNDC1 in CL. FUNDC1 mRNA expression (a) and (b), ROC (c) in patients with CL; FUNDC1 mRNA and protein expression (d) and (e) in leukemia cells. Normal, normal serum sample; Cancer, patients with CL; **p < 0.01 compared with normal serum sample or HS-5 cells.

To investigate the potential role of FUNDC1 in CL patients, we examined the expression levels of FUNDC1 using PCR. Our data revealed that the mRNA expression levels of FUNDC1 were up-regulated in serum samples from CL patients, compared to normal group (Figure 1a). Furthermore, the mRNA expression of FUNDC1 was lower in I–II stage CL patients compared to III–IV stage patients, with an receiver operating characteristic (ROC) value of 0.8536 (Figure 1b and c). Meanwhile, FUNDC1 mRNA and protein expression levels were also up-regulated in leukemia cell lines, compared with HS-5 cell (Figure 1d and e). We also observed an increase in FUNDC1 protein expression in 6T-CEM cells (Figure 1e). These findings suggest that FUNDC1 expression is increased in leukemia and may play a role in the progression of the disease.

### 3.2 FUNDC1 up-regulation promotes leukemia metastasis in *in vitro model* of leukemia

**Figure 2:**
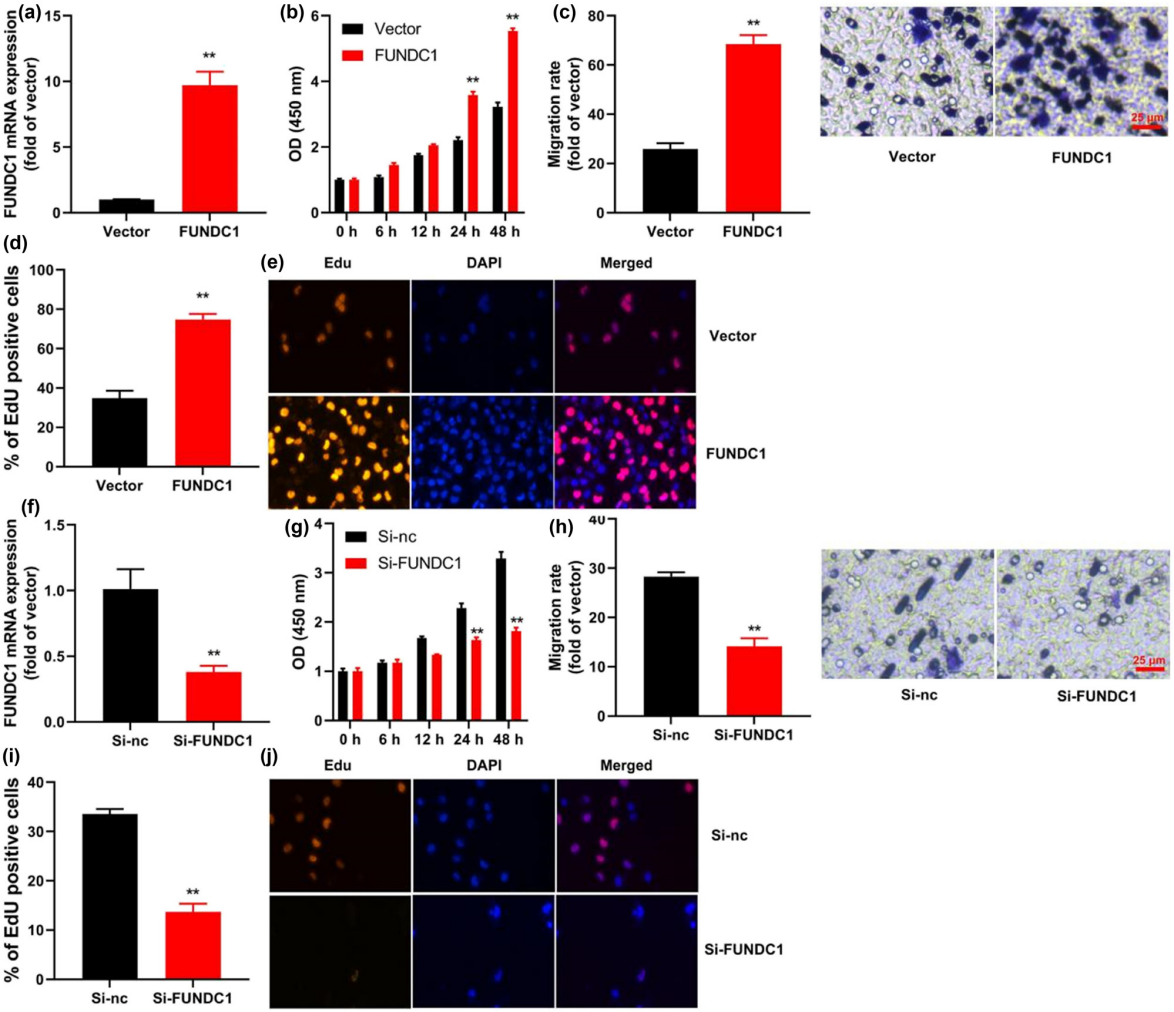
FUNDC1 up-regulation promotes leukemia metastasis. FUNDC1 mRNA expression (a), cell growth (b), metastasis (c), and EDU assay (d) *in vitro* model of leukemia by FUNDC1 up-regulation; FUNDC1 mRNA expression (e), cell growth (f), metastasis (g), and EDU assay (h) *in vitro* model of leukemia by FUNDC1 down-regulation. (i). Fluorescence images showing reduced proliferation in leukemia cells with FUNDC1 knockdown, as evidenced by fewer EDU-positive cells. (j). Micrographs illustrating decreased migration of leukemia cells after FUNDC1 knockdown. Vector, negative control group; FUNDC1, over-expression of FUNDC1 group; Si-nc, si-negative control group; Si-FUNDC1, down-regulation of FUNDC1 group; **p < 0.01 compared with negative control group or si-negative control group.

To investigate the role of FUNDC1 in leukemia cell metastasis, we conducted an experiment where we overexpressed FUNDC1 in leukemia cells using a FUNDC1 plasmid (Figure 2a). Our results demonstrated that up-regulation of FUNDC1 significantly enhanced cell proliferation, as indicated by an increase in EDU-positive cells, and increased the migration rate of leukemia cells (Figure 2b–d). On the other hand, when we knocked down FUNDC1 using siRNA, we observed a decrease in FUNDC1 mRNA expression in leukemia cells (Figure 2e), which subsequently inhibited cell proliferation, as indicated by a decrease in EDU-positive cells, and reduced the migration rate (Figure 2f–h). These findings suggest that up-regulation of FUNDC1 promotes cell proliferation and metastasis in leukemia. Further validation using fluorescence microscopy confirmed the reduction in cell proliferation under FUNDC1 knockdown conditions, as evidenced by fewer EDU-positive cells (Figure 2i). Additionally, microscopy images depicted reduced cellular migration, supporting the quantitative data (Figure 2j). These findings collectively suggest that the up-regulation of FUNDC1 promotes cell proliferation and metastasis in leukemia.

### 3.3 FUNDC1 up-regulation reduces ferroptosis by the inhibition of mitochondrial damage in *in vitro model* of leukemia

**Figure 3:**
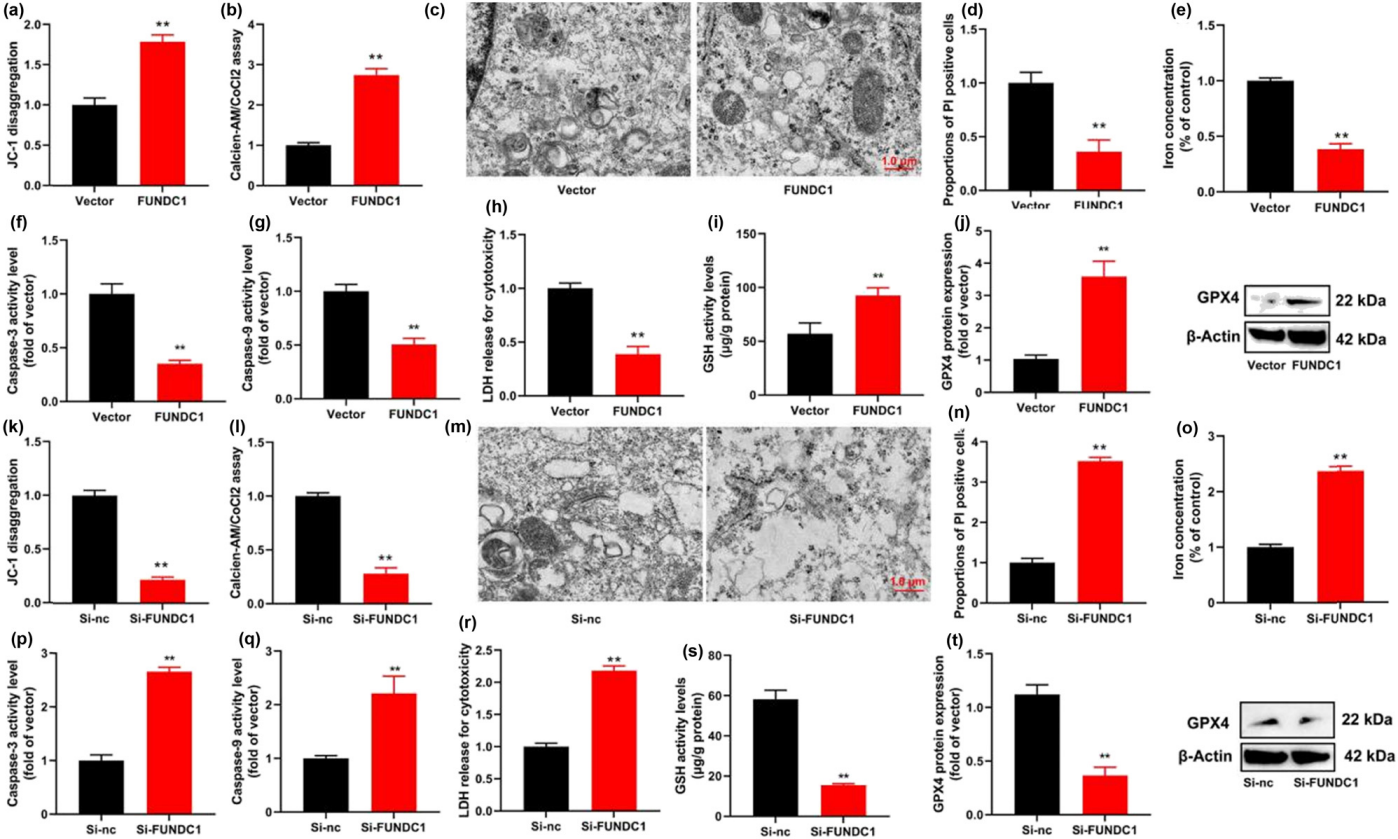
FUNDC1 up-regulation reduces ferroptosis by inducing mitochondrial damage. JC-1 levels (a), MPT (b), mitochondrial damage (c), PI-positive cells (d), iron content (e), caspase-3/9 activity (f) and (g), LDH activity levels (h), GSH levels (i), and GPX4 protein expression (j) *in vitro* model of leukemia by FUNDC1 up-regulation; JC-1 levels (k), MPT (i), mitochondrial damage (l), mitochondrial damage (m), PI-positive cells (n), iron content (o), caspase-3/9 activity (p) and (q), LDH activity levels (r), GSH levels (s), and GPX4 protein expression (t) *in vitro* model of leukemia by FUNDC1 down-regulation. Vector, negative control group; FUNDC1, over-expression of FUNDC1 group; Si-nc, si-negative control group; Si-FUNDC1, down-regulation of FUNDC1 group; **p < 0.01 compared with negative control group or si-negative control group.

In addition, we delved into the role of FUNDC1 in ferroptosis using an *in vitro* model of leukemia. Our findings revealed that the up-regulation of FUNDC1 led to an increase in JC-1 levels and mitochondrial permeability transition (MPT) as determined by the calcein AM/CoCl2 assay. It also resulted in a decrease in PI-positive cells and the activity levels of caspase-3/9. Moreover, FUNDC1 up-regulation inhibited the activity levels of lactate dehydrogenase (LDH) and the iron content in leukemia cells. It expanded the levels of glutathione (GSH) and increased the protein expression of GPX4 (Figure 3a–i). Conversely, knockdown of FUNDC1 reduced JC-1 levels and MPT, increased PI-positive cells and caspase-3/9 activity levels, promoted LDH activity levels and iron content, suppressed GSH levels, and decreased GPX4 protein expression in leukemia cells (Figure 3j–q). These data suggest that the up-regulation of FUNDC1 mitigates ferroptosis by inhibiting mitochondrial damage in leukemia cells.

### 3.4 FUNDC1 up-regulation induces FBXL2 expression in *in vitro model* of leukemia

**Figure 4:**
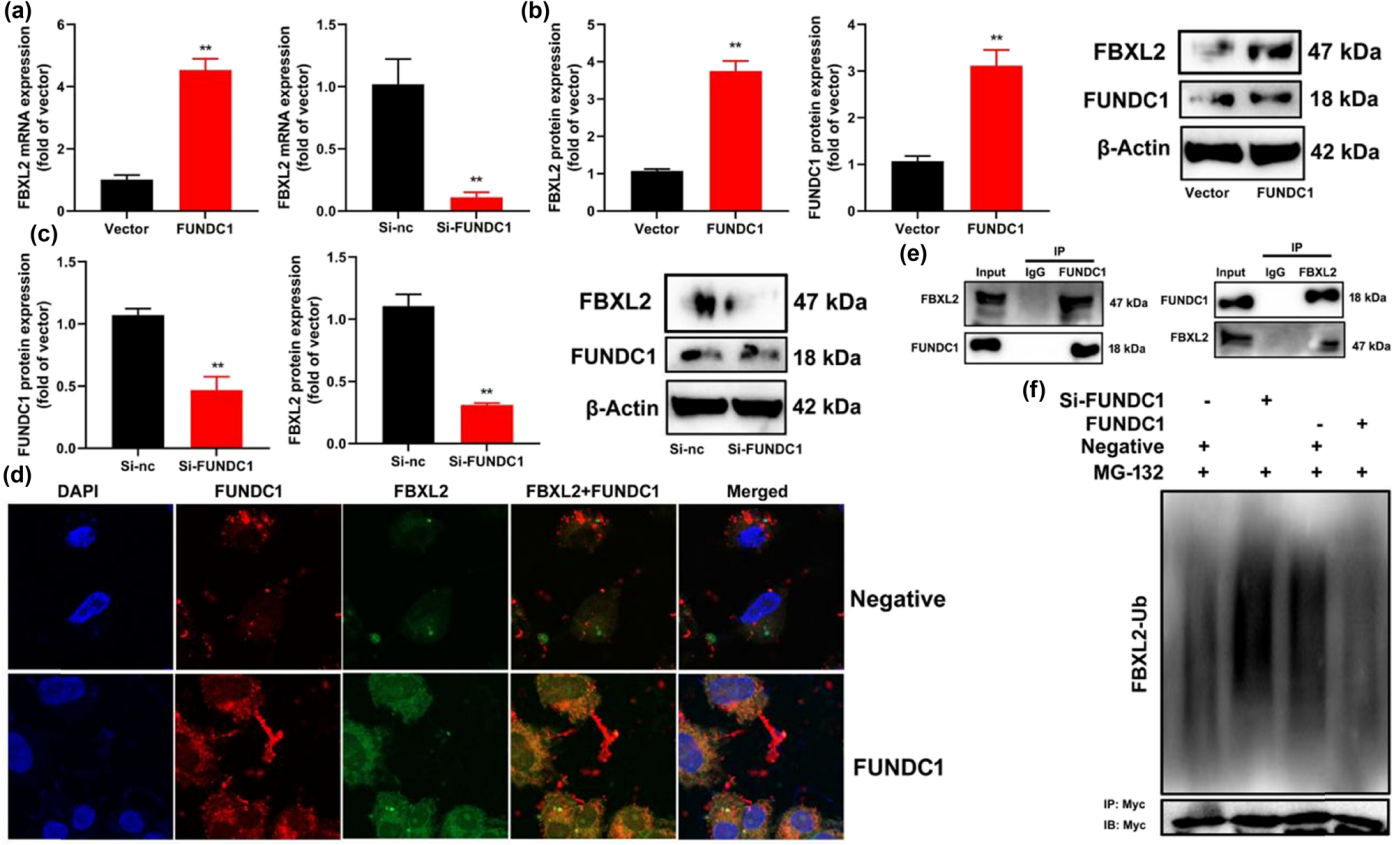
FUNDC1 up-regulation induces FBXL2 expression in a leukemia model. FBXL2 mRNA expression (a), FBXL2/FUNDC1 protein expression (b) and (c), FBXL2/FUNDC1 expression confocal microscope (d), FBXL2 protein interlinked with FUNDC1 protein (e), FBXL2 ubiquitination (f). Vector, negative control group; FUNDC1, over-expression of FUNDC1 group; Si-nc, si-negative control group; Si-FUNDC1, down-regulation of FUNDC1 group; **p < 0.01 compared with negative control group or si-negative control group.

The study further investigated the role of FBXL2 in mediating the effects of FUNDC1 on ferroptosis in leukemia through mitochondrial damage. We observed that the up-regulation of FUNDC1 resulted in an increase in FBXL2 mRNA expression, while si-FUNDC1 suppressed FBXL2 mRNA expression in leukemia cells (Figure 4a). Furthermore, FBXL2 up-regulation suppressed the protein expression of both FBXL2 and FUNDC1 in leukemia cells (Figure 4b), whereas FBXL2 down-regulation led to a decrease in the protein expression of FBXL2 and FUNDC1 (Figure 4c). Confocal microscopy analysis revealed that FUNDC1 up-regulation increased the expression of both FUNDC1 and FBXL2 in leukemia cells (Figure 4d). Additionally, immunoprecipitation experiments demonstrated that FBXL2 protein interacted with FUNDC1 protein (Figure 4e). Moreover, we found that the up-regulation of FUNDC1 reduced FBXL2 ubiquitination, thereby maintaining FBXL2 protein expression in leukemia cells (Figure 4f). Collectively, these findings suggest that FUNDC1 forms a complex with FBXL2 to attenuate FBXL2 ubiquitination in the leukemia model.

### 3.5 Mitochondrial damage regulates the effects of FUNDC1 up-regulation on ferroptosis and metastasis in *in vitro model* of leukemia

**Figure 5:**
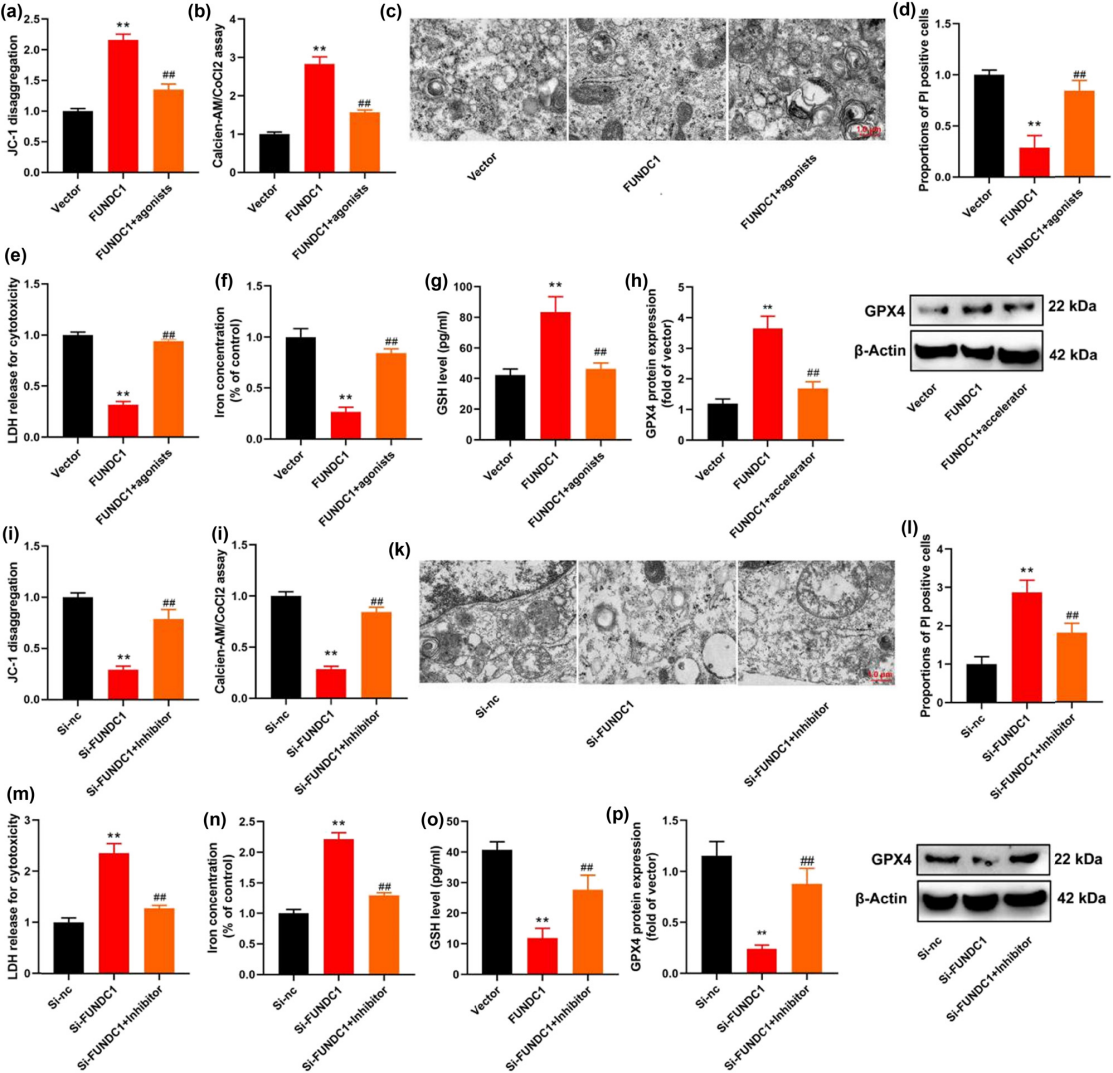
Mitochondrial damage regulates the effects of FUNDC1 up-regulation on ferroptosis in leukemia. JC-1 levels (a), MPT (b), mitochondrial damage (c), PI-positive cells (d), LDH activity levels (e), iron content (f), GSH levels (g), and GPX4 protein expression (h) *in vitro* model of leukemia by FUNDC1 up-regulation and activator; JC-1 levels (i), MPT (j), mitochondrial damage (k), PI-positive cells (l), LDH activity levels (m), iron content (n), GSH levels (o), and GPX4 protein expression (p) *in vitro* model of leukemia by FUNDC1 down-regulation and inhibitor. Vector, negative control group; FUNDC1, over-expression of FUNDC1 group; Si-nc, si-negative control group; si-FUNDC1, down-regulation of FUNDC1 group; **p < 0.01 compared with negative control group or si-negative control group; ^##^ *p* < 0.01 compared with FUNDC1 or si-FUNDC1 group.

**Figure 6:**
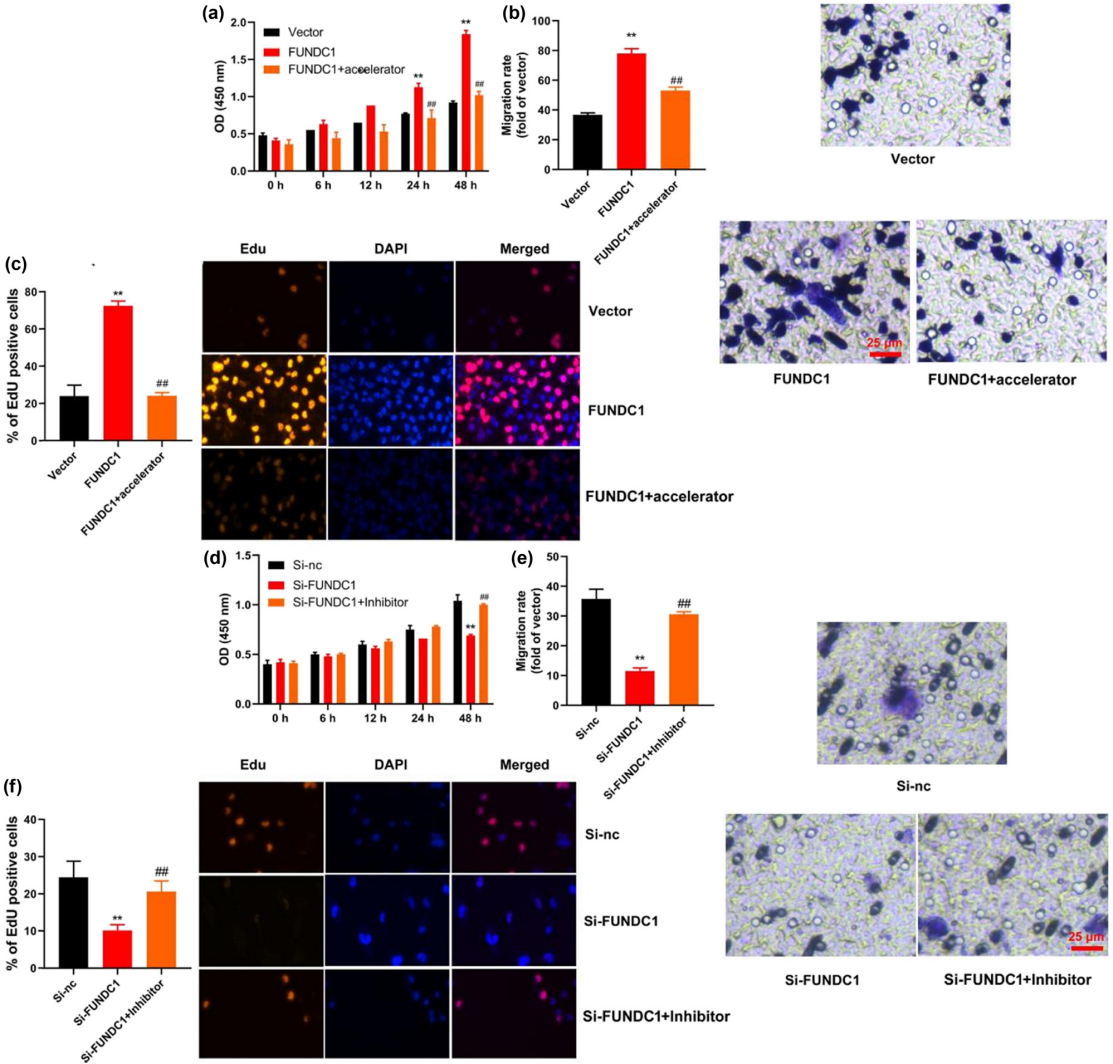
Mitochondrial damage regulates the effects of FUNDC1 up-regulation on metastasis in leukemia. Cell growth (a), metastasis (b), and EDU assay (c) *in vitro* model of leukemia by FUNDC1 up-regulation; cell growth (d), metastasis (e), and EDU assay (f) *in vitro* model of leukemia by FUNDC1 down-regulation. Vector, negative control group; FUNDC1, over-expression of FUNDC1 group; Si-nc, si-negative control group; Si-FUNDC1, down-regulation of FUNDC1 group; **p < 0.01 compared with negative control group or si-negative control group.

We investigated the functional involvement of mitochondrial damage in the effects of FUNDC1 on ferroptosis in leukemia. To induce mitochondrial damage, we treated leukemia cells with a mitochondrial damage activator, BMS-191095, at a concentration of 20 μmol/L. As a result, we observed a reduction in JC-1 level and MPT, an increase in PI positive cells and LDH activity level, and a decrease in GSH level and GPX4 protein expression in leukemia cells with FUNDC1 up-regulation (Figure 5a–h). Conversely, when we used a mitochondrial damage inhibitor, olesoxime, at a concentration of 10 µM, we observed an increase in JC-1 level and MPT, a decrease in PI positive cells and caspase-3/9 activity levels, a decrease in LDH activity level, and an increase in GSH level and GPX4 protein expression in leukemia cells with FUNDC1 down-regulation (Figure 5i–p). These findings suggest that mitochondrial damage is functionally involved in the effects of FUNDC1 on ferroptosis in leukemia. Next, we further investigated the impact of mitochondrial damage on leukemia cells with FUNDC1 up-regulation or down-regulation. Treatment with the mitochondrial damage activator led to a significant decrease in cell proliferation, as demonstrated by reduced EDU positive cells, and a decreased migration rate in leukemia cells with FUNDC1 up-regulation (Figure 6a–c). Conversely, treatment with the mitochondrial damage inhibitor resulted in enhanced cell proliferation, as indicated by increased EDU positive cells, and an increased migration rate in leukemia cells with FUNDC1 down-regulation (Figure 6d–f). These findings strongly suggest that FUNDC1 exerts its protective effect against ferroptosis in leukemia by inhibiting mitochondrial damage through the induction of FBXL2.

### 3.6 Methylation controls FUNDC1 stability in *in vitro model* of leukemia

**Figure 7:**
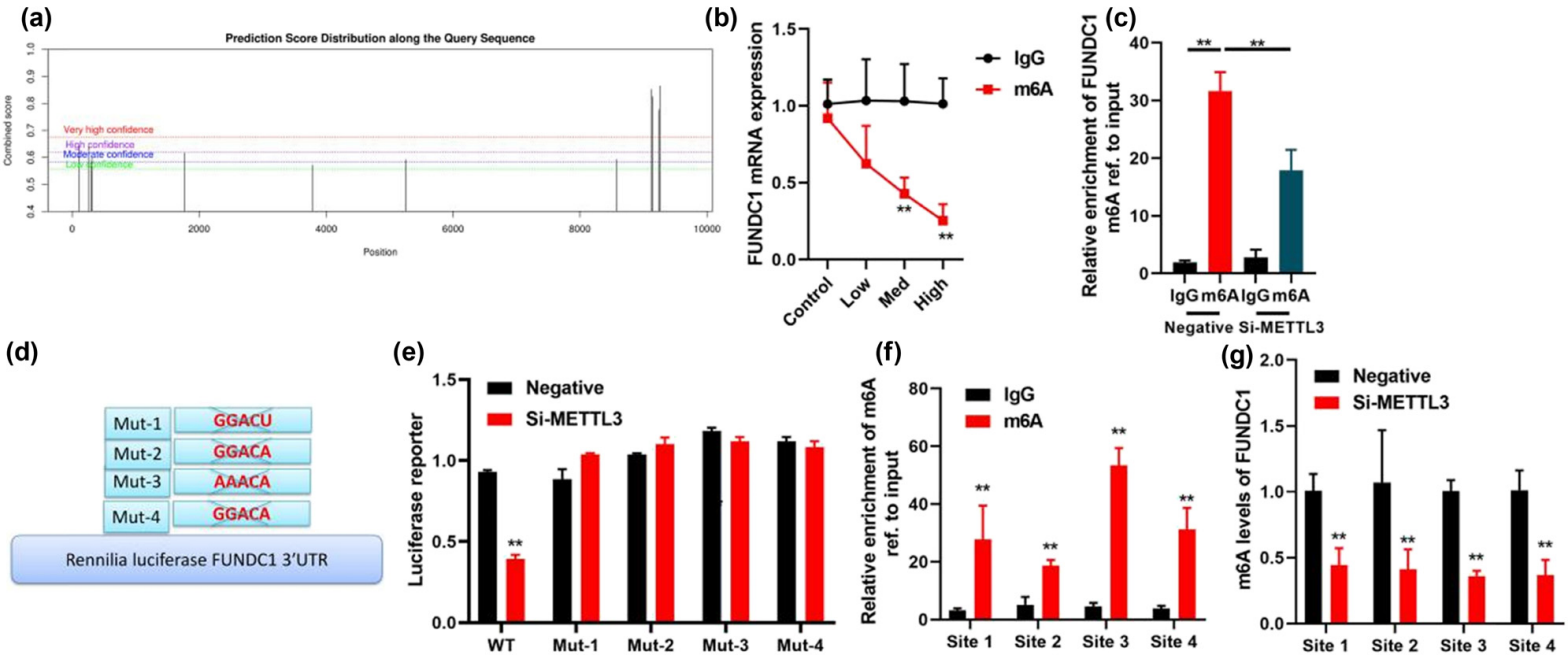
Methylation controls FUNDC1 stability. m6A modification site of FUNDC1 (a), METTL3-mediated FUNDC1 m6A modifications (b) and (c), the position of m6A motifs within FUNDC1 transcript sequence (d), luciferase reporter activity level (e), m6A levels of FUNDC1 (f) and (g), **p < 0.01 compared with vector or negative or IgG group.

The FUNDC1 gene contains several potential methylation modification sites near its stop codon, as shown in Figure 7a. *In vitro* models of leukemia treated with si-METTL3, an m6A antibody, resulted in a decrease in FUNDC1 mRNA enrichment levels (Figure 7b). Si-METTL3 also decreased the stability of FUNDC1 mRNA in these *in vitro* leukemia models (Figure 7c). Four m6A sites were identified in the 3ʹ-untranslated region of FUNDC1, with significant enrichment observed at sites 1, 2, 3, and 4 (Figure 7d). Si-METTL3 reduced the luciferase activity levels of wild-type FUNDC1 and two of its sites (Figure 7e). Furthermore, m6A enrichment increased the levels of FUNDC1 at sites 1, 2, 3, and 4 (Figure 7f), while si-METTL3 reduced the m6A levels of FUNDC1 at these sites (Figure 7g). These findings indicate that METTL3 methylation plays a role in controlling the stability of FUNDC1.

## 4 Discussion

Leukemia is the most prevalent cancer in children, accounting for approximately 35% of all childhood malignancies. It has become a chronic, non-communicable disease that poses a serious threat to children’s health [13,14]. CL differs significantly from adult leukemia in terms of disease spectrum composition, morphology, immunology, cytogenetics, molecular biology, and clinical prognosis. This indicates that CL has specific characteristics in terms of its pathogenesis, epidemiology, and other aspects [15]. In this study, the mRNA expression levels of FUNDC1 were up-regulated in serum samples from CL patients and leukemia cell lines. Hou et al. suggested that FUNDC1 might be a prognostic biomarker in patients with cervical cancer [16]. So, these results showed that FUNDC1 participated in disease progression of CL. In this study, we only collected 40 patients, which is insufficient, and is also the weakness of this study. We hope to collect more patients in the next experiment.

ALL is the most prevalent malignant hematological disorder among children in China, making up around 65–70% of all cases of childhood AL [17]. Childhood ALL is currently one of the malignancies with the most successful treatment outcomes and highest rates of cure, with a 5-year event-free survival rate exceeding 80% [18]. We found that FUNDC1 up-regulation promotes leukemia metastasis. Hui et al. showed that FUNDC1 regulated laryngeal cancer cells survival [19]. Meanwhile, this study only used cell lines, which is insufficient, we will use more models in further experiment.

Iron is a crucial metal ion involved in various metabolic processes in the human body and plays a significant role in promoting ferroptosis [20,21]. Exosomes released by cells treated with GPX4 inhibitors contain high levels of ferritin [20]. During ferroptosis, the level of prominin 2 is inversely correlated with the amount of free iron in the cell, suggesting that exosomes can protect cells from ferroptosis by eliminating iron from the cell [6]. Ferroptosis is accompanied by the accumulation of ROS and an increase in lipid peroxidation levels. GPX4 mitigates the susceptibility of cells to ferroptosis by reducing ROS accumulation through its antioxidant activity; consequently, the level and activity of GPX4 can influence the extent of ferroptosis [7]. Moreover, system X- plays a critical role in ferroptosis by regulating the ROS balance through its impact on GSH metabolism [22]. These data of this study showed that FUNDC1 reduces ferroptosis in leukemia through the inhibition of mitochondrial damage by the induction of FBXL2. Bi et al. favored that FUNDC1 interacts with GPX4 to trigger ferroptosis in model of liver fibrosis through mitochondrial translocation [23].

In conclusion, this study demonstrates that FUNDC1 promotes metastasis and reduces ferroptosis in a leukemia model through FBXL2 activation. These findings provide a new mechanism for understanding the inhibition of FUNDC1 in leukemia and suggest a novel target for cancer treatment. Therefore, FUNDC1 is a potential target for the treatment of various types of cancer in further clinical applications.

## References

[1] Makuuchi Y, Nakashima Y, Nishimoto M, Koh H, Hino M, Nakamae H. Posttransplant cyclophosphamide contributes to the impairment of the graft-versus-leukemia effect and the amelioration of graft-versus-host disease with the suppression of alloreactive T cells in a murine stem cell transplant model. Exp Hematol. 2023. 10.1016/j.exphem.2023.04.003. 37098360

[2] Cai Y, Chen X, Lu T, Yu Z, Hu S, Liu J, et al. Single-cell transcriptome analysis profiles the expression features of TMEM173 in BM cells of high-risk B-cell acute lymphoblastic leukemia. BMC Cancer. 2023;23(1):372. 10.1186/s12885-023-10830-5. PMC10123968 37095455

[3] Beneyto-Calabuig S, Merbach AK, Kniffka JA, Antes M, Szu-Tu C, Rohde C, et al. Clonally resolved single-cell multi-omics identifies routes of cellular differentiation in acute myeloid leukemia. Cell Stem Cell. 2023;30(5):706–721.e8. 10.1016/j.stem.2023.04.001. 37098346

[4] Catanzaro E, Turrini E, Kerre T, Sioen S, Baeyens A, Guerrini A, et al. Perillaldehyde is a new ferroptosis inducer with a relevant clinical potential for acute myeloid leukemia therapy. Biomed Pharmacother. 2022;154:113662. 10.1016/j.biopha.2022.113662. 36800294

[5] Grignano E, Cantero-Aguilar L, Tuerdi Z, Chabane T, Vazquez R, Johnson N, et al. Dihydroartemisinin-induced ferroptosis in acute myeloid leukemia: links to iron metabolism and metallothionein. Cell Death Discovery. 2023;9(1):97. 10.1038/s41420-023-01371-8. PMC10020442 36928207

[6] Sabatier M, Birsen R, Lauture L, Mouche S, Angelino P, Dehairs J, et al. C/EBPa confers dependence to fatty acid anabolic pathways and vulnerability to lipid oxidative stress-induced ferroptosis in FLT3-mutant leukemia. Cancer Discov. 2023;13(7):1720–47. 10.1158/2159-8290.cd-22-0411. 37012202

[7] Trombetti S, Iaccarino N, Riccio P, Sessa R, Catapano R, Salvatore M, et al. Over-expressed GATA-1(S), the short isoform of the hematopoietic transcriptional factor GATA-1, inhibits ferroptosis in K562 myeloid leukemia cells by preventing lipid peroxidation. Antioxidants (Basel). 2023;12(3):537. 10.3390/antiox12030537. PMC10045147 36978786

[8] Lyu T, Li X, Song Y. Ferroptosis in acute leukemia. Chin Med J (Engl). 2023;136(8):886–98. 10.1097/cm9.0000000000002642. PMC10278762 37010259

[9] Guo X, Zhou X. Risk stratification of acute myeloid leukemia: assessment using a novel prediction model based on ferroptosis-immune related genes. Math Biosci Eng. 2022;19(12):11821–39. 10.3934/mbe.2022551. 36653976

[10] Ponneri Babuharisankar A, Kuo CL, Chou HY, Tangeda V, Fan CC, Chen CH, et al. Mitochondrial Lon-induced mitophagy benefits hypoxic resistance via Ca(2+)-dependent FUNDC1 phosphorylation at the ER-mitochondria interface. Cell Death Dis. 2023;14(3):199. 10.1038/s41419-023-05723-1. PMC10020552 36927870

[11] Chen L, Zhang Q, Meng Y, Zhao T, Mu C, Fu C, et al. Saturated fatty acids increase LPI to reduce FUNDC1 dimerization and stability and mitochondrial function. EMBO Rep. 2023;24(4):e54731. 10.15252/embr.202254731. PMC10074135 36847607

[12] Ma L, Li K, Wei W, Zhou J, Li Z, Zhang T, et al. Exercise protects aged mice against coronary endothelial senescence via FUNDC1-dependent mitophagy. Redox Biol. 2023;62:102693. 10.1016/j.redox.2023.102693. PMC10113862 37030149

[13] Mendez-Hernandez A, Moturi K, Hanson V, Andritsos LA. Hairy cell leukemia: where are we in 2023? Curr Oncol Rep. 2023;25(8):833–40. 10.1007/s11912-023-01419-z. PMC10126561 37097545

[14] Obeidat M, Al-Khraisat IF, Jaradat DMM, Ghanim BY, Abdallah QM, Arqoub DA, et al. Mellitin peptide quantification in seasonally collected crude bee venom and its anticancer effects on myelogenous K562 human leukaemia cell line. BMC Complement Med Ther. 2023;23(1):132. 10.1186/s12906-023-03897-x. PMC10127481 37098530

[15] O’Connor D, Demeulemeester J, Conde L, Kirkwood A, Fung K, Papaleonidopoulou F, et al. The clinicogenomic landscape of induction failure in childhood and young adult T-cell acute lymphoblastic leukemia. J Clin Oncol. 2023;41(19):3545–56. 10.1200/jco.22.02734. PMC10306434 37098241

[16] Hou H, Er P, Cheng J, Chen X, Ding X, Wang Y, et al. High expression of FUNDC1 predicts poor prognostic outcomes and is a promising target to improve chemoradiotherapy effects in patients with cervical cancer. Cancer Med. 2017;6(8):1871–81. 10.1002/cam4.1112. PMC5548885 28719148

[17] Rieu JB, Canali A, Thene E, Tavitian S, Bertoli S. Acute promyelocytic leukaemia associated with atypical basophilia. Br J Haematol. 2023;201(6):1017. 10.1111/bjh.18793. 37096910

[18] Siamoglou S, Boers R, Koromina M, Boers J, Tsironi A, Chatzilygeroudi T, et al. Genome-wide analysis toward the epigenetic aetiology of myelodysplastic syndrome disease progression and pharmacoepigenomic basis of hypomethylating agents drug treatment response. Hum Genomics. 2023;17(1):37. 10.1186/s40246-023-00483-7. PMC10127336 37098643

[19] Hui L, Wu H, Wang TW, Yang N, Guo X, Jang XJ. Hydrogen peroxide-induced mitophagy contributes to laryngeal cancer cells survival via the upregulation of FUNDC1. Clin Transl Oncol. 2019;21(5):596–606. 10.1007/s12094-018-1958-5. 30284230

[20] Zhong FM, Yao FY, Liu J, Zhang HB, Zhang J, Zhang N, et al. Ferroptosis-related molecular patterns reveal immune escape, inflammatory development and lipid metabolism characteristics of the tumor microenvironment in acute myeloid leukemia. Front Oncol. 2022;12:888570. 10.3389/fonc.2022.888570. PMC9742468 36518303

[21] Wang X, Li Q, Sui B, Xu M, Pu Z, Qiu T. Schisandrin a from schisandra chinensis attenuates ferroptosis and NLRP3 inflammasome-mediated pyroptosis in diabetic nephropathy through mitochondrial damage by adipor1 ubiquitination. Oxid Med Cell Longevity. 2022;2022:5411462. 10.1155/2022/5411462. PMC9391610 35996380

[22] Yu Y, Meng Y, Xu X, Tong T, He C, Wang L, et al. A ferroptosis-inducing and leukemic cell-targeting drug nanocarrier formed by redox-responsive cysteine polymer for acute myeloid leukemia therapy. ACS Nano. 2023;17(4):3334–45. 10.1021/acsnano.2c06313. 36752654

[23] Bi Y, Liu S, Qin X, Abudureyimu M, Wang L, Zou R, et al. FUNDC1 interacts with GPx4 to govern hepatic ferroptosis and fibrotic injury through a mitophagy-dependent manner. J Adv Res. 2024;55:45–60. 10.1016/j.jare.2023.02.012. PMC10770120 36828120

